# Regulation of Cell Death by Mitochondrial Transport Systems of Calcium and Bcl-2 Proteins

**DOI:** 10.3390/membranes10100299

**Published:** 2020-10-21

**Authors:** Natalia Naumova, Radek Šachl

**Affiliations:** J. Heyrovský Institute of Physical Chemistry, Czech Academy of Sciences, 182 23 Prague, Czech Republic; naumova.kandaurova@gmail.com

**Keywords:** mitochondria, calcium transport, Bcl-2 proteins, apoptosis, necrosis, Bax, MCU, VDAC, mPTP

## Abstract

Mitochondria represent the fundamental system for cellular energy metabolism, by not only supplying energy in the form of ATP, but also by affecting physiology and cell death via the regulation of calcium homeostasis and the activity of Bcl-2 proteins. A lot of research has recently been devoted to understanding the interplay between Bcl-2 proteins, the regulation of these interactions within the cell, and how these interactions lead to the changes in calcium homeostasis. However, the role of Bcl-2 proteins in the mediation of mitochondrial calcium homeostasis, and therefore the induction of cell death pathways, remain underestimated and are still not well understood. In this review, we first summarize our knowledge about calcium transport systems in mitochondria, which, when miss-regulated, can induce necrosis. We continue by reviewing and analyzing the functions of Bcl-2 proteins in apoptosis. Finally, we link these two regulatory mechanisms together, exploring the interactions between the mitochondrial Ca^2+^ transport systems and Bcl-2 proteins, both capable of inducing cell death, with the potential to determine the cell death pathway—either the apoptotic or the necrotic one.

## 1. Introduction

Calcium (Ca^2+^) is a divalent cation and a universal second messenger that regulates the most important functions and facets of all eukaryotic cells, including gene expression, proliferation, regulation of bioenergetics, contraction of muscles, mediation of fertilization, and many other cellular functions [1,2,3,4,5]. Regulation of free intracellular concentration of Ca^2+^ is an important mechanism for intracellular signaling, and it is a key component in the mediation of many cell functions and biochemical reactions, being crucial for signal transduction in cells [2,6,7,8,9,10]. On top of all that, intra-mitochondrial Ca^2+^ regulates a cascade of physiological and pathophysiological processes in cells [10,11,12,13,14,15].

The organelles responsible for Ca^2+^ homeostasis are undoubtedly the mitochondria, which are essential for cellular bioenergetics by storing energy in the form of ATP and by playing a major role in Ca^2+^ signaling [5,12,16,17]. Ca^2+^ uptake by mitochondria not only participates in the regulation of cytosolic Ca^2+^ concentration ([Ca^2+^]), but also stimulates mitochondrial respiration and ATP production [18,19]. These properties make these organelles the major cellular components in the regulation of the fate of a cell [9,12,17,20,21].

Localization of mitochondria inside the cell can vary significantly: from the periphery of the cell, around the nucleus, but also close to the plasma membrane or the endoplasmic/sarcoplasmic reticulum (ER/SR) [22,23]. These different localizations determine the Ca^2+^-buffering capacity of each individual mitochondria, as well as the mitochondrial network [12]. Upon contact of the mitochondria, or more specifically the outer mitochondrial membrane (OMM) with other organelles, membrane contact sites are formed [24]. These inter-organelle associations have various functions. For instance, those formed between the mitochondria and ER/SR (mitochondria-associated membranes, MAMs) determine Ca^2+^-uptake from the cytoplasm to the mitochondria, and therefore play an essential role in the Ca^2+^ signaling pathways [23,25,26]. It is established that such associations contain microdomains with high Ca^2+^ concentrations that determine the mediation of Ca^2+^ transport between the mitochondria and the ER/SR [24]. Moreover, mitochondrial associations with the plasma membrane are engaged in the mediation of Ca^2+^ transport from the extracellular environment [27].

Mitochondria are the power generators of cells. They produce ATP in the citric acid cycle (the tricarboxylic acid (TCA) or the Krebs cycle (see Box 1 for more information). Production of ATP involves activation of the Ca^2+^-dependent dehydrogenases in the citric acid cycle, F0F1-ATP-synthase and metabolite transporters; all of them being supplied by basal oscillating increases in the concentration of Ca^2+^ in the mitochondrial matrix [6,28,29,30]. In addition to these normal physiological oscillations, large Ca^2+^ spikes in mitochondria can cause an opening of the mitochondrial permeability transition pore (mPTP) [9,28,29]. In turn, this induces a collapse of the mitochondrial membrane potential, termination of oxidative phosphorylation processes, osmotic changes, mitochondrial swelling, and inner membrane remodeling. All of these processes culminate by mitochondrial outer membrane permeabilization (MOMP) and the release of cytochrome c; being both an inducer of apoptosis and modulator of other proapoptotic factors [2,12,31,32]. Whereas mostly associated with programmed cell death, a number of compounds trigger changes in Ca^2+^ homeostasis and mPTP-induced apoptosis [2,9,29].

Box 1 **The generation of ATP by mitochondria.**

Mitochondria are the power generators within all eukaryotic cells. They release their energy in the form of ATP by the oxidation of sugars. Electrons supplied by NADH are transferred to oxygen by a series of protein complexes in the inner mitochondrial membrane. By pumping protons across the membrane, these complexes create a transmembrane electrochemical gradient (ΔΨ, ~−180 mV). This reverse current of protons into the mitochondrial matrix occurs through a proton channel formed by ATP synthase, and it is used to store energy in the form of ATP.

The ability of mitochondria to uptake and retain Ca^2+^ had already been described in the early 1960s using isolated mitochondria [33,34]. During the same years, the chemiosmotic theory, as proposed by Mitchell [35], revealed the thermodynamic basis of the process.

**Figure 1:**
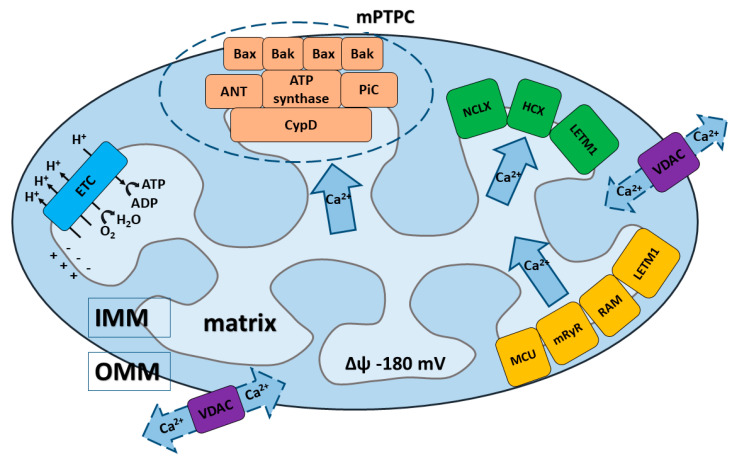
Schematic presentation of Ca^2+^ transport systems in mitochondria. (1) Ca^2+^ influx and efflux through the outer mitochondrial membrane (OMM) driven via the voltage-dependent anion channel (VDAC). (2) Ca^2+^ influx through the inner mitochondrial membrane (IMM) driven by three major transport systems: (i) mitochondrial Ca^2+^ uniporter (MCU), (ii) mitochondrial ryanodine receptor (mRyR), (iii) rapid mode of Ca^2+^ uptake (RaM) and one mitochondrial system under debate: (iv) leucine zipper- EF-hand containing transmembrane protein (LETM1). Ca^2+^ influx through the MCU is established by the electrochemical gradient created by the electron transport chain (ETC). (3) Ca^2+^ efflux through the IMM driven by three other major transport systems: (i) Na^+^/Ca^2+^/Li^+^ (NCLX) exchanger, (ii) H^+^/Ca^2+^ exchanger (HCX), (iii) mitochondrial permeability transition pore complex (mPTPC) and one mitochondrial system under debate: (iv) leucine zipper- EF-hand containing transmembrane protein (LETM1). (4) The core constituents of mPTPC include: the adenine nucleotide translocase (ANT), matrix cyclophilin D (CypD) and phosphate carrier (PiC), which serve as pore regulators, and the pro-apoptotic proteins Bax and Bak, which can induce mitochondrial swelling and rupture during the mPTP opening. ATP-synthase is the key IMM-pore forming unit of mPTPC.

Mitochondria are able to rapidly accumulate and transiently store Ca^2+^ for later quick release, making these organelles important cytosolic depots or buffers for Ca^2+^ regarding mediation of the cell’s physiological and pathological processes, including from cell survival to cell death [3,7,9,12,21,36,37]. Regulated elevations of Ca^2+^ levels in the mitochondrial matrix are necessary for the regulation of Ca^2+^-dependent mitochondrial enzyme activity, which sequentially mediates the metabolic balance and function of the mitochondrial electron transport chain, as well as the production of mitochondria-generated reactive oxygen species (ROS) [12,38,39,40]. Undoubtedly, the precise regulation of mitochondrial Ca^2+^ uptake and release is necessary for proper cellular functioning and regulation of mitochondrial bioenergetics. The normal level of intra-mitochondrial Ca^2+^ is essential for the correct functioning of mitochondria, whereas Ca^2+^ overload is typical for a wide range of mitochondrial dysfunctions and pathophysiological processes [14,15,37,39,41]. Homeostasis of Ca^2+^ in the mitochondria is determined by the delicate balance of mitochondrial Ca^2+^ transport systems in both the inner (IMM) and outer mitochondrial membrane (OMM) (Figure 1). Ca^2+^ influx and efflux systems are composed of different components, including: channels, pumps, antiporters, or Ca^2+^ binding proteins that cooperate to maintain intra-mitochondrial Ca^2+^ homeostasis [10,14,38,39,42].

## 2. Calcium Transport Systems in Mitochondria

### 2.1. Calcium Influx and Efflux through OMM

When Ca^2+^ enters the mitochondrial matrix from the cytoplasm, it first encounters the OMM. This membrane is highly permeable to cations, anions, and molecules with molecular weights < 5 kDa due to the presence of large conductance channels. These channels, formed by voltage-dependent anion channel proteins (VDACs), allow for the exchange of Ca^2+^ and small molecules by concentration gradients [2,43,44,45,46,47]. They not only regulate transport of Ca^2+^ from the cytoplasm into the intermembrane space (IMS), but are additionally engaged in the mediation of cellular metabolism by transporting ATP and other small metabolites across the OMM [12,48]. Importantly, the permeability of VDACs is precisely controlled and regulated, particularly by ATP and a variety of cellular regulatory factors.

VDAC (Figure 1) was the first channel that has been reconstituted and characterized in detail at the single-channel level [44,49]. It has been proposed to work as the principal metabolite transport system across the OMM, and had also been proposed to serve as the interconnection point between the OMM and IMM [50]. Later, three different VDAC isoforms were identified: VDAC1, VDAC2, and VDAC3 [2,44,47,51].

VDAC1 is highly expressed in most cells, and seems to be the most prevalent and most extensively characterized; it is also considered as the main transport channel for Ca^2+^ [2,52]. VDAC1 is the gatekeeper for the passage of ions and metabolites, and is crucial for the regulation of apoptosis, thanks to its interactions with pro- and anti-apoptotic proteins [23,53]. Activity of VDAC1 is critical for the mitochondrial metabolic pathways balance, as well as for cell survival [53]. Imaging of VDAC1 by stimulated emission depletion nanoscopy revealed the organization of VDAC proteins into clusters in H9C2 cells, which has also been studied in VDAC transfected U2OS cells [54,55]. VDAC1 consists of 19 transmembrane β-strands that are organized into the membrane-incorporated β-barrel and a amphipathic 26-residue-long N-terminal domain, which can translocate from the pore interior to the channel surface [56]. This behavior is crucial for controlling the gating of the channel as well as its interactions with apoptotic proteins [56,57]. Whereas isoforms of VDAC1 and VDAC2 self-assemble into structures resembling a pore, VDAC3 forms smaller conductance channels that are able to modulate the physiological functions of various proteins [58]. As demonstrated by Checchetto et al. [58], VDAC3 isoforms demonstrate different electrophysiological properties compared with those of VDAC1 and VDAC2. In the context of their structural/functional characteristics, VDAC1, VDAC2, and VDAC3 have some similarities; at the same time, they exhibit different physiological functions regarding their interaction with cytosolic proteins and other mitochondrial proteins [2,47,57,59]. Furthermore, only limited information is available regarding the potential functions of VDAC2 and VDAC3 for the influx of Ca^2+^ [43,59,60].

### 2.2. Calcium Influx through IMM

Compared to the OMM, the IMM exhibits a fundamentally higher selectivity for anions and cations thanks to the presence of highly-specific and different protein machinery in the IMM. The key transporters that determine Ca^2+^ uptake by mitochondria through the IMM until recently were unclear. It is now believed that the transport of Ca^2+^ through the IMM is accomplished by a group of mitochondrial Ca^2+^ uptake transporters. Basically, three main mechanisms of Ca^2+^ influx have been proposed (Figure 1): (1) a mechanism that requires an electrogenic mitochondrial Ca^2+^ uniporter multi-protein complex (MCU complex); (2) a so-called rapid mode (RaM); (3) a mechanism requiring the mitochondrial ryanodine receptor (mRyR) [7,10,12,13,38]; and (4) additionally, leucine zipper-EF-hand containing transmembrane protein (LETM1) could represent another Ca^2+^ influx mechanism, but its role is still under discussion [61,62,63,64].

#### 2.2.1. Calcium Influx by Mitochondrial Ca^2+^ Uniporter (MCU) Multi-Protein Complex

The molecular identity of this Ca^2+^ transport pathway had been unclear for several decades. However, in 2011, the CCDC109a gene, a pore-forming component of the MCU channel, mediating Ca^2+^ influx into mitochondria was discovered [65,66]. The protein encoded by the CCDC109a gene is responsible for Ruthenium Red-sensitive mitochondrial Ca^2+^ uptake. Currently, accumulation of Ca^2+^ through the MCU multi-protein complex is the most widely characterized and commonly accepted pathway of Ca^2+^ influx into mitochondria; and it is considered as the major pathway of the mitochondrial Ca^2+^ influx. It is determined by a large electrochemical gradient (~−180 mV) across the IMM, and may be inhibited by Ruthenium Red and lanthanides [7,13,42,67,68,69]. The complex consists of several subunits, including transmembrane core components and regulatory subunits that are associated with the membrane. The core components of the MCU multi-protein complex (see Box 2 for details) are comprised of: (a) core protein components: Mitochondrial Ca^2+^ Uniporter (MCU), a MCU dominant negative beta subunit (MCUb), and Essential MCU REgulator (EMRE); and (b) membrane associated regulatory components: mitochondrial Ca^2+^ uptake protein 1–3 (MICU1–3) and Mitochondrial Ca^2+^ Uniporter Regulator 1 (MCUR1) [12,13,23,42,47,68,69,70,71,72]. Solute Carrier 25A23 (SLC25A23)) was initially identified as an essential component of MCU, however, it is currently under debate whether SLC25A23 is an component of MCU or whether it influences MCU indirectly [13,42,73]. Importantly, the MCU complex can be found in multiple states.

Box 2 **Structure of the MCU multi-protein complex.**

Core components

**MCU (mitochondrial Ca^2+^ uniporter, previously known as CCDC109a, 40 kDa)** is a key core component of the complex. It is encoded by a highly conservative MCU gene and is present in virtually all eukaryotic organisms [10,13,47]. MCU can be found in multiple states, and it consists of two coiled-coil domains (CC) and two transmembrane domains connected via a short loop (9 amino acid residues) containing a highly conserved DIME motif [42,65,66].

**MCUb (MCU dominant negative beta subunit, formerly known as CCDC109b, 40 kDa)** is a core component of the MCU multi-protein complex encoded by the MCUb gene, and is present in all vertebrates [71,72,74]. It exhibits a 50% homology with MCU; however, MCU and MCUb demonstrate diverse expression profiles in different tissues. Importantly, MCUb significantly impairs Ca^2+^ permeation through MCU [42,69,70].

**EMRE (essential MCU regulatory element, 10–12 kDa)** is the last core component identified in the complex. It contains a single transmembrane segment, and crucially regulates MCU activity as has been shown using EMRE knockout cells, which inhibited mitochondrial Ca^2+^ influx [75]. EMRE is assumed to be involved in the formation of interactions between the core and the regulatory subunits, despite the fact that such ensembles of regulatory components do not require the presence of EMRE [13,42,47,75].

Membrane-associated regulatory components

**MICU1 (mitochondrial Ca^2+^ uptake protein 1, known as CBARA1/EFHA3, 54kDa)** known as CBARA1/EFHA3, is a membrane-associated and water-soluble component localized in the inter-membrane space; it is considered as central for the activation of MCU. In the resting state (i.e., at low intracellular concentrations of Ca^2+^), MICU1 blocks access of Ca^2+^ to the MCU channel [75,76,77,78]. It also acts as a cooperative activator of MCU and it stimulates MCU Ca^2+^-transport conductivity [76].

**MICU2 (mitochondrial Ca^2+^ uptake protein 2, known as EFHA1, 50 kDa)** and **MICU3 (mitochondrial Ca^2+^ uptake protein 3, known as EFHA2, 60 kDa)** display the EF-hand domains in the protein structure, and were identified as MiCU1 paralogs with 41% and 34% identity to the MICU1, respectively [78,79,80]. MiCU2 forms heterodimers with MiCU1 through disulfide bonds, and acts as a Ca^2+^ sensor, protecting the mitochondria against Ca^2+^ overload, and it also acts as the regulator of several cell functions [76,81,82].

**MCUR1 (mitochondrial Ca^2+^ uniporter regulator 1, known as CCDC90A, 40 kDa)** is composed of 2 transmembrane domains and 1 specific coiled-coil region, and it belongs to yet another regulatory component of the MCU complex [83,84]. MCUR1 knockdown prevents Ca^2+^ entry into the mitochondria; whereas, its overexpression promotes mitochondrial Ca^2+^ uptake [82,84]. MCUR1 interacts with EMRE and MCU-pore via its coiled-coil domains, which stabilize all components of the MCU complex [85]. It is involved in the assembly of the mitochondrial respiratory chain, and represents a cytochrome c oxidase assembly factor; possibly also regulating the mitochondrial membrane potential [86].

**SLC25A23 (solute carrier 25A23, 48–54 kDa)** was initially identified in the IMM as a protein with the EF-hand domain, and has been proposed as a component of MCU multi-protein complex [2,86,87]. SLC25A23 may also function as an ATP-Mg/Pi exchanger, promoting the influx of adenine nucleotides into the matrix of mitochondria and the efflux of inorganic phosphate. Of note, SLC24A23 functions in a Ca^2+^ dependent manner [73,88]. Mutations and modifications of the EF-hand domains in this carrier decrease Ca^2+^ influx into mitochondria; however, it still remains unclear whether SLC25A23 influences the uniporter complex directly or whether it affects the mitochondrial bioenergetics [13,42,87]. Further studies are necessary to understand the exact mechanism by which SLC25A23 regulates mitochondrial Ca^2+^ influx.

#### 2.2.2. Rapid Mode Mechanism (RaM) of Ca^2+^ Uptake

The RaM (RApid Mode of Ca^2+^ uptake) mechanism is able to accumulate Ca^2+^ up to a hundred times faster compared with the MCU multi-protein complex (no molecular structure responsible for this mechanism has yet been identified) [89,90]. It is transiently activated by low calcium concentrations (50–100 nM) and by high concentrations of Ruthenium Red [13,90,91]. This behavior contrasts sharply with MCU, which is activated by Ca^2+^ concentrations higher than 500 nM. RaM promotes mitochondria to rapidly sequester Ca^2+^ at the beginning of each cytosolic Ca^2+^ pulse, and rapidly recovers between pulses, allowing mitochondria to respond to repetitive Ca^2+^ oscillations [13,91]. It is still speculated that RaM is just an additional state of the MCU multi-protein complex because of their similarity as well as the absence of RaM in MCU knockout mitochondria [65,90,91]. At present, the progress of research targeted on explaining the role of RaM in Ca^2+^ influx at the molecular level is very limited.

#### 2.2.3. The Mechanism of Ca^2+^ Uptake Requiring Mitochondrial Ryanodine Receptor (mRyR)

mRyR (mitochondrial ryanodine receptor, 600 kDa) is the ryanodine-sensitive mitochondrial Ca^2+^ uptake mechanism, capable of Ca^2+^ transport, which was detected in the IMM of isolated heart mitochondria in 2001 by Beutner at al. [92]. This group confirmed the presence of the ryanodine receptor in the IMM using [3H]ryanodine binding, RyR antibody conjugated immunogold particles, and Western blot analysis [92]. It could serve as an alternative mechanism for Ca^2+^ accumulation in mitochondria as well as a regulator of Ca^2+^ efflux under mitochondrial Ca^2+^ overload and pathological conditions [92,93,94]. Interestingly, the single channel activity of mRyR was confirmed on recombinant mRyR proteins reconstituted in supported lipid bilayers prepared from IMM vesicles [95]. This study elucidates pharmacological and electrophysiological features of mRyR in the model of IMM merged to lipid bilayers, where a mitochondrial transporter with gating properties similar to those of RyR in ER/SR was demonstrated [95].

#### 2.2.4. The Mechanism of Ca^2+^ Uptake Including LETM1

LETM1 (leucine zipper- EF-hand containing transmembrane protein, 70 kDa) is an integral mitochondrial inner membrane protein, usually co-localized with a mitochondrial matrix protein HSP60 [62,96]. The N-terminus of this protein is linked to the IMM via a transmembrane domain consisting of 3 proline residues, whereas the C-terminus extends to the mitochondrial matrix [62,63,97]. It was also demonstrated previously that LETM1 is an endogenous protein in HeLa cells, with a molecular weight of 83 kDa, and it has been assumed that it is initially produced as a cytosolic precursor with a presequence [62,96,98,99]. LETM1 is a transporter protein shown to exhibit Ca^2+^/H⁺ exchange activity, acting as a crucial component in the regulation of Ca^2+^ homeostasis [62,96,100,101,102,103,104]. Later it was proposed as an inner mitochondrial membrane Ca^2+^/H^+^ antiporter [103] that is able to transport Ca^2+^ bidirectionally across the membrane. In addition, experimental work indicated the important role of LETM1 in maintaining K^+^ homeostasis, and this has led to the suggestion that LETM1 works as an H^+^/K^+^ exchanger with an electroneutral activity (1H^+^/1K^+^) [105]. Of note, this exchanger shares a key role with MCU to catalyze Ruthenium Red-sensitive transport of Ca^2+^ into mitochondria [103]. It would likely serve as an alternative mechanism for Ca^2+^ accumulation in mitochondria, as well as a regulator of Ca^2+^ efflux under mitochondrial Ca^2+^ overload [61,101,106]. In summary, although the importance of LETM1 for cellular functioning is clear, the molecular characteristics and details of LETM1 organization still remain unclear.

### 2.3. Calcium Efflux through IMM

In order to maintain the intra-mitochondrial Ca^2+^ homeostasis under physiological and pathological conditions, the balance between Ca^2+^ influx and efflux into/from mitochondria has to be maintained. The functional and molecular characterization of the mitochondrial Ca^2+^ efflux system already had started in the 1970s, when Na^+^-dependent Ca^2+^ efflux from mitochondria was described in isolated rat heart mitochondria [107], and two different mechanisms were proposed: (1) Na^+^-dependent (Na^+^/Ca^2+^/Li^+^ exchange, NCLX) and (2) Na^+^-independent (H^+^/Ca^2+^ exchange, HCX) mechanisms. It was reported that the Na^+^/Ca^2+^ exchange takes place in excitable tissues (i.e., brain, heart), whereas H^+^/Ca^2+^ exchange is typical for non-excitable tissues (i.e., liver). However, both systems provide slow Ca^2+^ release in comparison to the rate of Ca^2+^ influx through the MCU [108,109]. Later, (3) the mitochondrial permeability transition pore complex (mPTPC) was identified as an important Ca^2+^ efflux mechanism [3,110]. Besides this, LETM1 (4) has been proposed as an additional Ca^2+^ efflux system [12,103,111,112] (Figure 1).

#### 2.3.1. The Mechanism of Ca^2+^ efflux by NCLX

NCLX (Na^+^/Ca^2+^/Li^+^ exchanger systems): Mitochondrial Na^+^/Ca^2+^ (NCX) exchange was discovered by Carafoli et al. in 1974 [107]. However, the molecular composition of the Na^+^-dependent Ca^2+^ efflux system was resolved relatively recently [113], and interestingly, seems to function as a transporter of Li^+^ ions as well, being a member of the family of Na^+^/Ca^2+^ exchangers [73,113,114,115]. The ability of NCLX to conduct both Na^+^/Ca^2+^ and Li^+^/Ca^2+^ transport is a unique feature of the mitochondrial carrier [73,115,116]. In fact, it can transport either Li^+^ or Na^+^ in exchange for Ca^2+^. NCLX is the only known member of the Na^+^/Ca^2+^ exchanger superfamily that can also transport Li^+^ [73,115]. Na^+^/Ca^2+^ exchangers are characterized as transporters with a low affinity and high capacity; thus, they could be most effective in regulating of Ca^2+^ homeostasis during transient Ca^2+^ fluxes commonly expressed in excitable cells [116,117].

NCLX mechanism predominates in the mitochondria of cardiomyocytes, neurons, cells of the skeletal muscle, parotid gland, adrenal cortex, and brown fat [115,118,119] and to a lesser extent also being present in lung mitochondria and mitochondria of the kidney and liver [2,67,120]. NCLX can be inhibited by benzodiazepines and CGP37157 inhibitor of the mitochondrial Na^+^/Ca^2+^ exchanger [121,122]. Of note, under conditions when mitochondria are depolarized, all types of Ca^2+^ exchangers can act in the reverse mode, pumping Ca^2+^ into the mitochondria [13,123].

#### 2.3.2. The Mechanism of Ca^2+^ Efflux by HCX

HCX (H^+^/Ca^2+^ exchanger): Na^+^-independent Ca^2+^ efflux (HCX) is prevalent in mitochondria of non-excitable cells (i.e., liver, kidney, lung, smooth muscles), in contrast to the NCLX mechanism [16,67,94,119,124,125]. The molecular composition of the HCX is still unclear and the literature on this complex sparse; however, it is assumed to be electroneutral with the stoichiometry of 2 molecules of H^+^ per 1 molecule of Ca^2+^ [125,126]. The rate of Ca^2+^ efflux through HCX decreases with an increase in the pH gradient [124,126].

#### 2.3.3. The Mechanism of Ca^2+^ Efflux by LETM1

LETM1 (leucine zipper- EF-hand containing transmembrane protein, 70 kDa): In comparison to NCX, NCLX, or HCX, Ca^2+^ efflux via LETM1 does not represent the major pathway, but it could serve as an alternative mechanism for the release of Ca^2+^ [2,12,103,111,112,119]. Moreover, the activity of this protein might be essential for maintenance of the tubular shape of mitochondria and for cristae organization [9,96]. In addition, LETM1 can work as a Ca^2+^/H^+^ antiporter (see Section 2.2.4—The Mechanism of Ca^2+^ Uptake Including LETM1) [127].

#### 2.3.4. The Mechanism of Ca^2+^ Efflux by mPTP/mPTPC

mPTP/mPTPC (mitochondrial permeability transition pore or mPTP complex): mPTP or mPTPC is considered as the main transport system for Ca^2+^ efflux from mitochondria under pathophysiological conditions [2,3,12,31,32,128,129,130,131,132,133,134]. Although the mPTPC was initially described in swelling experiments using the fraction of isolated mitochondria and characterized as a non-selective channel that transports ionic and nonionic molecules as early as 1979 [110], the transport mechanism of this channel actually remains poorly understood.

It is commonly believed that mPTPC is a multi-protein system in the OMM and IMM. Originally, only regulatory components were identified. The first unambiguously established component was CypD (Figure 1), which still remains the only protein whose involvement in mPTPC pore formation and activity regulation is undisputed [135,136,137,138,139]. CypD can stimulate structural rearrangements in the proteins responsible for the formation of mPTPC pore channel, preventing mPTP-mediated necrosis [13,136]. Most of the studies on the role of CypD in the regulation of mPTP relied on pharmacological cyclosporin A or transient siRNA inhibition of CypD, as well as on the results obtained on models of the knockout mouse, which demonstrated its interconnection with mPTPC [135,137,138,140].

Adenine nucleotide translocase (ANT) was initially believed to represent the main regulatory component of mPTPC [141]. Recent studies characterized ANT as a pore-forming component and proposed a “multi-pore model” with two separate pore-forming molecular components: one of which is ANT and the other depends on CypD [142]. It is also possible that CypD and ANT function in a “dual regulatory model”, where mPTPC is regulated by both ANT and CypD [142]. Moreover, it is currently believed that ANTs are multifunctional proteins, which represent not only the pore-forming component of the mPTPC but may also be crucial for mitochondrial uncoupling and for the stimulation of mitophagy [143].

Furthermore, F0F1 ATP Synthase and the phosphate carrier (PiC) are considered as the core pore-forming components of mPTPC [130,144,145,146,147,148]. FoF1 ATP Synthase forms the channel in mPTC and transports molecules through the 2 ATP synthase monomers or through the ring of the c-subunit, which overlaps with the IMM and the pore forming component [148,149]. However, it should be noted that classification of the last named component (PiC) is more complicated, since in the context of its ability to activate mPTP opening it can be considered as the pore forming component [150]. At the same time, following patch clamping of the PiC displayed too low of a conductance to assume that it functions as the core pore-forming constituent of the mPTPC. Undoubtedly, the precise nature and molecular organization of the pore-forming part of mPTPC remain controversial [130,148,149,151,152,153,154,155,156,157,158,159,160,161]. m-AAA protease Spastic Paraplegia 7 (SPG7) was previously thought to be a core component of the mPTP that is able to interact with CypD and with VDAC1 at the OMM/IMM contact sites [136]. However, recent results demonstrate that SPG7 is not a core component of the mPTP, but could regulate the mPTP activity by decreasing Ca^2+^ levels in mitochondrial matrix through modulation of MCUR1 and MCU assembly [146].

The efflux of Ca^2+^ occurs through a transient or low conductance opening of mPTP, most likely by lower oligomeric states of mPTP [13,131,162,163,164,165,166,167,168]. The evidence for transient opening of mPTP for Ca^2+^ was demonstrated by the early studies on the inhibition of Ca^2+^ release by Cyclosporin A in isolated adult rat ventricular cardiomyocytes [133]. Transient opening or low conductance opening of the mPTP represent a Ca^2+^ efflux mechanism, and various studies have confirmed the essential role of mPTP in the release of Ca^2+^ [13,131,164,165,166,167,168]. mPTP is a nonspecific channel, used by cells in signal transduction and the transfer of molecules between the mitochondrial matrix and cytoplasm. In particular it maintains Ca^2+^ homeostasis, regulates oxidative stress signals, and mediates cell death [128,131,169,170]. Regarding the multi-conductance function of mPTPC, it likely can be assumed that mPTPC is partially oligomerized into a complex with multiple subunits [132,170,171]. The first studies using different sized polyethylene glycols identified solutes of up to 1500 Da that could be transported through the pore that matches the modeled pore size of 1.4 nm [110]. Importantly, mPTP is able to reversibly open upon an increase in ADP concentration, as well as during restoration of the Mg^2+^/Ca^2+^ ratio [110], reestablishing mitochondrial membrane potential, and allowing for mPTP to have either a sustained or transient opening [132,172]. The different regimes of mPTP opening determine the selectivity in signaling.

The opening of mPTP is directly regulated by the concentration of free Ca^2+^, and triggered by mitochondrial Ca^2+^ overload, allowing for rapid Ca^2+^ release from mitochondria [3,13,32,70,132,156,173]. Obviously, Ca^2+^ is the most important regulator and inductor of mPTP opening, regarding its numerous indirect roles in the regulation and modulation of the mPTP [130,131,132,174]. The functional dualism of Ca^2+^ is an important factor of mPTP mediation. At physiological levels of Ca^2+^ it can activate transient opening of the pore; whereas at Ca^2+^ overload it can induce pathological changes, resulting in sustained mPTP opening and subsequent mitochondrial and cellular dysfunction [128,132,134,173,175].

Activation of mPTP could also be mediated at different levels through regulation by kinases, as well as posttranslational modification of CypD [176]. It has been shown, that mPTP could be stimulated by Ca^2+^ in combination with an increase in the concentration of ROS and phosphate; additionally, that it could be inhibited by divalent cations (such as Mg^2+^, Mn^2+^), adenine nucleotides, low pH, or CypD inhibitors (such as CsA and sanglifehrin A) [177]. Importantly, modifications and loss of CypD induce a significant increase in the threshold concentration of Ca^2+^ required for pore opening [13,136].

Hypothetically, VDAC could also mediate mPTPC activity; however, genetic analysis did not prove to be any essential function of this protein in mPTP-mediated cell death [53,175]. Electrophysiological and biochemical studies supported the molecular model of mPTPC with the VDAC on the OMM, ANT on the IMM, and CypD in the matrix [178,179,180]. In brief, the following facts speak for involvement of VDAC1 in mPTP opening and function: overexpression of microRNA-7 prevents opening of mPTP by downregulating VDAC1 [181]; the loss of mitochondrial fission factor Mff inhibits mPTP opening via blocking of VDAC1 oligomerization and separation of HKII, which leads to the inhibition of mPTP opening [1,182]. On the other hand, additional studies have provided opposing results, indicating that the closed state of VDAC stimulates Ca^2+^ permeability, and therefore forces mPTP opening [183,184].

## 3. The Family of Bcl-2 Proteins

**Figure 2:**
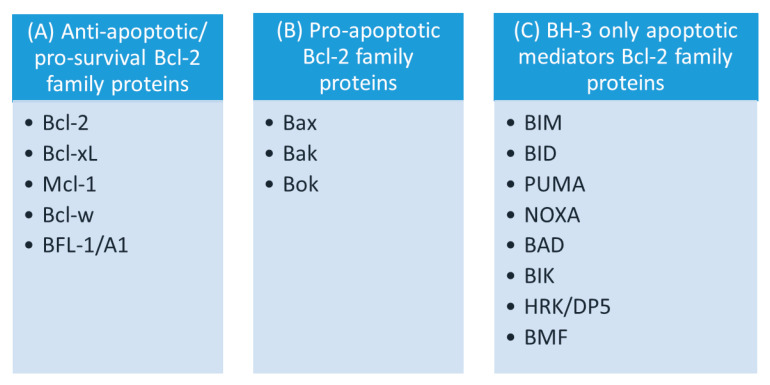
Classification of Bcl-2 of proteins based on their apoptotic activity. (**A**) anti-apoptotic; (**B**) pro-apoptotic; and (**C**) apoptotic mediators of the Bcl-2 family proteins containing one BH-3 domain.

The Bcl-2 (B-cell lymphoma-2) family of proteins represents a collection of pro- and anti-apoptotic proteins, functioning in a delicate balance during homeostasis (Figure 2). The main role of this group of proteins is the regulation of caspase activity and the execution of cells as a consequence of different intracellular and extracellular stimuli, and thereby dictating the fate of a cell [185,186,187,188,189]. They work as regulators of cell cycle progression, or autophagy, as well as the mediators of Ca^2+^ concentration, unfolded protein response and metabolism of the lipids and carbohydrates [169,185,190,191]. Importantly, Bcl-2 proteins are crucial regulators of Ca^2+^ homeostasis and dynamics due to their ability to directly target and regulate the intracellular Ca^2+^-transport systems, in particular IP3 receptors and mitochondrial Ca^2+^ transport systems [192,193,194].

Members of the Bcl-2 group of proteins arose during metazoan evolution [195,196], and they are characterized by the existence of short conserved sequence regions, the so-called Bcl-2 homology (BH) domains [197]. Up to now, four different BH domains have been described, and are numbered according to their date of discovery [198]. The first protein to be discovered was the Bcl-2 protein, first observed at the chromosome translocation breakpoints of leukemic cells and follicular lymphoma [199,200,201]. Bcl-2 members are typically α-helical globular proteins composed of 9 helices, although most BH3-only proteins are largely unstructured [202]. All proteins from this family share a common BH3 amino acid sequence, which is present in both anti-apoptotic and pro-apoptotic Bcl-2 homologs, as well as in a separate group of BH3-only proteins. BH3-containing helices regulate various of their physical interactions, which result either in cell survival or in one of the types of cell death. These properties have recently led to the development of therapeutic BH3 mimetics [188,189,191,203,204,205].

The Bcl-2 family comprises numerous members, categorized into 3 groups according to the total number of BH domains and their role in apoptosis (Figure 2): (1) pro-survival (i.e., anti-apoptotic) Bcl-2 proteins; (2) pro-cell death (pro-apoptotic) Bcl-2 proteins; and (3) BH3-only proteins [3,169,187,206,207,208,209,210,211].

### 3.1. Anti-Apoptotic Bcl-2 Proteins

The subfamily of pro-survival Bcl-2 proteins includes proteins with anti-apoptotic activity: Bcl-2 itself, Bcl-XL (Bcl-extra long), Mcl-1 (myeloid cell leukemia-1), Bcl-w (Bcl-2-like protein 2), and BFL-1/A1 (Table 1). This subfamily contains BH1, BH2, and BH3, and in some cases also a BH4 domain [211,212,213,214]. These members are generally integrated within the mitochondrial membrane, but are also present in other organelles (such as the endoplasmic reticulum). Furthermore, they are found in many cell types and tissues, where co-expression of multiple pro-survival proteins often occur [214,215]. Typically, they are anchored in the OMM by their α9 helix, and exposed to the cytoplasm by their globular Bcl-2 fold [202]. The lead member Bcl-2 (around 26 kDa) and its homologue Bcl-XL (around 27 kDa) are characterized by the presence of all four BH domains, and exert their anti-apoptotic role mainly through the involvement of BH1, BH2, and BH3 homology domains that mediate the interactions with the pro-apoptotic Bcl-2 members, preventing MOMP and subsequent apoptosis [188,211,216]. Another member, Mcl-1 (around 37 kDa), exhibits several unique features. First, it undergoes rapid degradation in response to different stress stimuli that may shift the threshold for induction of apoptosis. Second, it displays its own binding-affinity profile [217,218]. Mcl-1 sequesters both BIM and tBID activators, plus the putative PUMA and NOXA activators, as well as pore-forming proteins (Bak and Bax) [218,219,220].

### 3.2. Pro-Apoptotic Bcl-2 Proteins

The subfamily of pro-apoptotic Bcl-2 proteins contains: Bax (Bcl-2-associated X protein), Bak (Bcl-2-antagonist/killer-1) directly promoting MOMP, and Bok (Bcl-2 related ovarian killer), which is crucial for embryonic development; however, its role in cell death is as yet unclear. Proteins from this subfamily contain all four BH domains (Figure 2) [221,222,223]. Corresponding to the anti-apoptotic members of the Bcl-2 group of proteins, various pro-apoptotic proteins are present in cells and are involved in the regulation of apoptosis [215]. Bax (around 21 kDa) was the first identified pro-apoptotic member and an inhibitory binding partner of Bcl-2 [224].

Both Bax and Bak (around 28 kDa) are directed at the OMM by a C-terminal tail anchor [225]. It is proposed that Bak, like other C-terminal tail anchor proteins, is constitutively bound to its target—the mitochondrial membrane. In contrast, it has been shown that Bax is not normally bound to the mitochondrial membrane [226,227]. Bax is predominantly a monomer within the cytosol of nonapoptotic cells, with its C-terminal tail anchor sequence folded back and hidden within a hydrophobic groove on the protein’s surface [228]. However, recent results have shown that Bax is constitutively targeted to mitochondria during the apoptotic process and translocated back to the cytosol during normal cellular functioning [227]. It is now believed that Bax translocation between cytosol and mitochondria is a constitutive and dynamic process, and that Bax is in a dynamic balance between the cytosol and the membrane [215,227].

The pro-apoptotic function of Bax and Bak is activated in response to a range of cell death factors, causing them to undergo conformational changes, membrane-insertion, oligomerization, and the formation of a channel in the OMM [188]. Permeabilization of the mitochondrial membrane leads to the release of cytochrome c, caspase activation, and cell death [188]. Another member of this subfamily Bok (around 23 kDa) is characterized as the homolog of Bax and Bak, and it is involved in the regulation of apoptotic processes. It was demonstrated that mice with the Bok deficiency have a normal phenotype; however, enforced Bok expression could induce apoptosis in a wide range of cell types [215,229,230]. Moreover, it is still unclear whether Bok involvement in the regulation of apoptosis requires Bax or Bak [214,231].

### 3.3. BH3-Only Bcl-2 Proteins

The subfamily of BH3-only Bcl-2 proteins represents a heterogeneous group, which with the exception of BID contains proteins with only one highly conserved BH3 domain: BIM (Bcl-2-like 11), BID (BH3 interacting domain death agonist), PUMA (p53-upregulated modulator of apoptosis), NOXA (phorbol-12-myristate13-acetate-induced protein 1), BAD (Bcl-2-associated agonist of cell death), BIK (Bcl-2 interacting killer), HRK (Harakiri)/HRK/DP5 (Bcl-2 interacting protein death protein 5), and BMF (Bcl-2 modifying factor) (Figure 2) [3,209,210,212,231].

Upregulation of BH3-only proteins can occur at transcriptional/post-translational levels in response to stress, and this may result in cell death [215]. For example, PUMA (ca. 22 kDa) and NOXA (ca 11 kDa) are transcriptional targets of the p53 tumor suppressor, and their expression is increased in response to cytotoxic stimuli that activate p53 [232]. BID (ca 22 kDa) bears a BH3-motif with a folded α-helical structure [233,234]. During apoptosis, BID is cleaved by caspase-8 or other compounds including other caspases, granzyme B, calpains, or cathepsins [234]. Protease-cleaved Bid migrates to the mitochondria where it induces MOMP, which is dependent on the presence of Bax and/or Bak. Thus, BID acts as a sentinel for protease-mediated death signals [197,235].

### 3.4. Interactions between Bcl-2 Proteins

Various competing models describe the interactions of Bcl-2 proteins during the mediation of MOMP [187,210,236,237]. In all models, the BH3 domain is essential for proper functioning of the proteins, to a large extent controlled by mutual interactions of the proteins in intracellular membranes [189,238,239,240]. The most accepted model for the regulation of apoptosis by Bcl-2 proteins suggests heterodimeric interactions between Bcl-2 proteins playing an important role in the activation of Bax and Bak [3,241,242]. A specific subset of BH3-only proteins named “activators” (including BID, BIM, PUMA, and NOXA) directly bind and activate Bax and Bak, or inhibit pro-survival proteins; thereby inducing a series of their conformational changes, subsequent homo- and hetero-oligomerization within the OMM, and finally MOMP [3,189,218,240,241,242,243,244]. In turn, MOMP allows for the release of mitochondrial apoptogenic factors into the cytosol (including cytochrome c), SMAC (second mitochondria-derived activator of caspase), and DIABLO (direct IAP binding protein with low pI), HtrA2 (high temperature requirement protein A2), as well as others, which then initiate apoptosome formation and the induction of the caspase cascade [17,218,236,242].

Other members of the BH3-only proteins group most likely act as “sensitizers”. They are not the direct activators of Bax or Bak; however, they are able to bind Bcl-2 proteins with pro-survival functions [3,240]. The BH3 activator cBID sequestered by Bcl-XL complexes only changes from an inactive to an active form while bound to a Bcl-XL complex (and when Bad is also bound). Bcl-XL complexes enable Bad to function as a non-competitive inhibitor of Bcl-XL and allosterically activate BID, dramatically enhancing the pro-apoptotic potency of BAD [185,189,241]. Both the activators and sensitizers could be activated via specific mechanisms, leading to the transfer of the apoptotic stimuli to Bax and Bak, as well as to the pro-survival Bcl-2 proteins [3,242].

Interestingly, Bcl-2 proteins have a significant impact on the dynamic processes related to mitochondrial fusion and fission in cells [218,245,246,247,248,249]. The mitochondrial apoptotic pathway is based on cooperation between Bcl-2 proteins and other proteins found in mitochondria, and it is intimately related with membrane fission, thereby linking mitochondrial dynamics to apoptosis [24,247,250,251,252]. These processes are closely related to the functioning of the Ca^2+^ transport systems. For instance, it was demonstrated that Bax, Bak, and Bcl-XL induce changes in mitochondrial dynamics by regulating the activity of the proteins responsible for fusion and fission [246,248,249,253]. More specifically, it has been reported that Bax and Bak are involved in the control of mitochondrial fusion in different cell models (primary mouse neurons, mouse embryonic fibroblasts, and human colon carcinoma cells) through binding to the mitofusins—Mfn1 and Mfn2 [249]. It has also been shown that Bax or Bak are required in healthy cells for normal fusion of mitochondria into elongated tubules. Nevertheless, despite these interesting findings, the exact molecular mechanisms underlying the (de)activation of the mitochondrial dynamic machinery by Bcl-2 proteins still remain to be elucidated [245].

## 4. Regulation of Mitochondrial Ca^2+^ Transport Systems by Bcl-2 Proteins

Apart from the ability to influence mitochondrial permeability directly, Bcl-2 proteins are also involved in the mediation of apoptotic signaling by regulating Ca^2+^ homeostasis [29,185,193,254]. This regulation can occur through direct interactions between the intracellular Ca^2+^-transport systems and anti-apoptotic Bcl-2 members; in particular Bcl-2, Bcl-XL, Mcl-1, Bax, and Bak [255,256,257,258,259,260,261]. These members target crucial intracellular Ca^2+^-transport systems such as Ca^2+^-efflux systems of ER/SR (inositol 1,4,5-trisphosphate receptors (IP3Rs) and ryanodine receptors (RyRs)) and the Ca^2+^-efflux system of mitochondria (involving VDAC, which provides Ca^2+^ flux trough OMM, and mPTP, which represents the main Ca^2+^ efflux system from mitochondria under pathophysiological conditions) [193,194,211,262].

In general, the Bcl-2 proteins are major regulators of a wide range of mitochondrial functioning processes. Those involving regulation of mitochondrial dynamics and morphology have already been mentioned in chapter 3.4. Other processes are responsible for Ca^2+^ homeostasis and include regulation of the metabolism and functioning of the electron transport chain, permeability transition, and mPTP opening [185,193,194,254,255,263]. A substantial part of these processes is summarized in Table 1 for clarity.

### 4.1. Bcl-2 Proteins and Ca^2+^ Influx through VDAC

VDAC1 is a key component in the apoptotic pathway mediated by mitochondria, and is a target for pro- and anti-apoptotic Bcl-2 proteins [263,265,266]. The molecular composition of VDAC1 determines its mobility, which is necessary for controlling the channel gating and the interactions with pro- and anti-apoptotic proteins [56,256,263,266,267].

It was demonstrated that Bcl-2 proteins are able to directly target VDAC, and in turn modulate its activity [255]. These interactions mediate changes in a large number of mitochondrial processes, and may also regulate Ca^2+^ transport in mitochondria [268,269]. VDAC1 is considered as a key protein in the mPTP-mediated apoptosis by associating with pro- and anti-apoptotic members of the Bcl-2 family and hexokinase, and promotes the release of apoptotic proteins [268]. It has been shown that when the VDAC1 channel closes, it becomes impermeable to ATP and metabolites, but the permeability for Ca^2+^ increases [270]. This confirms the critical role of VDAC1 in mitochondrial Ca^2+^ homeostasis and the induction of cell death. On top of that, VDAC1 regulates translocation of pro-survival Ca^2+^ signals from the cytosol and ER/SR, as well as the ATP transport and metabolite exchange between the cytosol and mitochondria [193]. Furthermore, pro-survival proteins (e.g., hexokinase-I) are required for the correct functioning of VDAC1 and Ca^2+^ signal transduction to mitochondria (also see Section 2.1) [271,272,273].

Considering all these VDAC1 roles in Ca^2+^-determined cell functioning and death it can be expected that Mcl-1, Bcl-XL, and Bcl-2 are able to emulate the interactions between VDAC1 and Ca^2+^ transport in mitochondria (Table 1). For instance, it has been shown that Bcl-2 and Bcl-XL bind directly to the N-terminus of VDAC1, thereby inhibiting mitochondrial Ca^2+^ uptake and protecting cells from extreme mitochondrial uptake of Ca^2+^ [256,266]. Similarly, it was shown that BH4 oligopeptides of Bcl-2 and Bcl-XL inhibit VDAC1 activity on liposomes and apoptotic depolarization of isolated mitochondria [254]. Turning to the mechanism by which this could happen, it has been suggested that Bcl-XL promotes the open configuration of VDAC1 [274] and the BH4 domain of Bcl-XL selectively targets one to two VDAC1 molecules [260,269,275,276]. Despite these findings, a study that comparing wild-type MEF cells versus Bcl-XL-gene deficient MEF cells demonstrated that the presence of Bcl-XL promoted the accumulation of Ca^2+^ in the matrix by increasing Ca^2+^ transfer across the OMM [269]. As was demonstrated by electrophysiological studies, Bcl-XL could stimulate either the increase [274] or the decrease [276] in VDAC1 conductance. Interestingly, structural-functional studies of Bcl-XL demonstrate that induction of Ca^2+^ influx into mitochondria by Bcl-XL is related to the formation of Bcl-XL dimers [277]. Formation of these dimers is facilitated by the recruitment of Bcl-XL to the membrane, and is inhibited by BH3 mimetics [277]. From this point of view, Bcl-XL is able to enhance the activity of enzymes involved in the Krebs cycle, and can therefore stimulate mitochondrial bioenergetic processes in healthy nonapoptotic cells. This means that Bcl-XL could function as a direct anti-apoptotic protein capable of enhancing transduction of the Ca^2+^ signals to the mitochondria from the cytosol and ER/SR, and mediate Ca^2+^ oscillations that are crucial for both ATP synthesis and the bioenergetic balance [193,278].

Another key anti-apoptotic member of the Bcl-2 family that is capable of direct binding to VDAC channels is Mcl-1 [257]. It was reported that Mcl-1 associates with high affinity to VDAC1 and VDAC3 isoforms, but not to VDAC2 [257]. In A549 cells, reduction of Mcl-1 expression levels lowered Ca^2+^ uptake into the mitochondrial matrix and inhibited generation of ROS. It has been demonstrated that in A549, H1299, and H460 cells Mcl-1 knockdown and VDAC derived peptides decreased cell migration, but that cell proliferation remained unchanged. Of note, cell migration could be restored in Mcl-1 knockdown cells by increasing the levels of ROS (following the experimental model in which migration is activated by the production of ROS). These data suggest that an interaction between Mcl-1 and VDAC promotes cell migration by a mechanism that involves Ca^2+^-dependent ROS production [257].

Furthermore, there is genetic and pharmacologic evidence that the BID and BAD pro-apoptotic factors also participate in the influx of Ca^2+^ by close cooperation with Bcl-XL and Bcl-2 [194] (Table 1). More specifically, the mitochondrion-localized isoform of Bcl-XL could induce cell migration by interacting with VDAC1 to mediate Ca^2+^ transport to mitochondria from the ER/SR and other Ca^2+^ depots in a BH3-dependent manner.

### 4.2. Bcl-2 Proteins and Na^+^/Ca^2+^ Exchangers

Although there is no evidence for direct interaction between a member of the Bcl-2 family and the Na^+^/Ca^2+^ exchangers family, it was shown already in 2001 that Bcl-2 could inhibit Ca^2+^ transport via the Na^+^/Ca^2+^ exchanger [264] (Table 1). More specifically, Bcl-2 overexpression in transgenic mice induced reduction of Na^+^/Ca^2+^ exchange and promoted the resistance to the permeability transition. This behavior was accompanied by increased Ca^2+^ concentration and the induction of transport proteins that could regulate the apoptotic pathway [264]. Despite these reports supporting the regulation of Na^+^/Ca^2+^ exchangers by Bcl-2 proteins, this research needs to be continued.

### 4.3. Bcl-2 Proteins and mPTP (See Also Section Calcium Efflux through mPTPC)

Direct protein–protein interactions between Bcl-2 proteins and proteins involved in mPTPC formation (ANT, ATP synthase, and VDAC) have been documented [32,255,261] (Table 1). In particular, the pro-apoptotic Bcl-2 proteins Bax and Bak have been reported to interact directly with mPTPC [252,258]. Both Bax and Bak participate in mPTPC mediation because of their ability to stimulate and increase MOMP, which allows mPTP opening followed by mitochondrial swelling [255,258,261]. It was also demonstrated that Bcl-2 could positively modulate ANT activity, whereas Bax inhibits ANT function by destabilizing the interactions with Bcl-2 [279]. The following findings that support a close relationship between Bcl-2 and mPTPC were observed in cells/mice deficient in Bax and Bak: (1) mPTP-induced cell death and related mitochondrial damage is inhibited in cardiac cells after a myocardial infarction [259]; (2) mitochondria are resistant to the opening of mPTP; (3) reconstitution of Bax in such cells restored both the mPTP opening [258,259] and outer mitochondrial membrane permeability [258]; and (4) Bax/Bak deficiency is protective regarding the conditions of ischemia-reperfusion injury [258,259]. According to expectations, Bcl-2 members with anti-apoptotic activity could also influence mPTPC by functioning through the interactions with regulatory components [274,280,281]. Further investigations are needed to clarify the molecular mechanism of mPTP regulation by Bax and Bak; nevertheless, recent studies demonstrate the importance of mitochondrial lipid composition for their ability to permeabilize the mitochondrial membrane [282].

In summary, it is evident that Bcl-2 proteins regulate the activity of the proteins and multiprotein complexes of mitochondrial Ca^2+^-transport systems, including VDAC, Na^+^/Ca^2+^ exchangers, and mPTPC. These interconnections influence regulation of Ca^2+^ homeostasis in mitochondria and in the cell, and mediate mitochondrial cell death pathways.

## 5. Regulation of Cell Death by Mitochondrial Transport of Calcium and Bcl-2 Proteins

The Nomenclature Committee on Cell Death 2018 classified cell death types regarding their biochemical characteristics, and following this protocol there are 12 different types of regulated cell death (RCD) (see Box 3 for more details) [283]. Additionally, programmed cell death (PCD) that takes place during physiological development and tissue turnover is classified as a physiological form of RCD [283].

Box 3 **The cell death.**

The 12 different types of the regulated cell death have so far been classified: necroptosis, ferroptosis, pyroptosis, parthanatos, entotic cell death, NETotic cell death, lysosome-dependent cell death (LDCD), autophagy-dependent cell death (ADCD), immunogenic cell death (ICD), intrinsic apoptosis, extrinsic apoptosis, and mitochondrial permeability transition (MPT)-driven (mediated) necrosis.

The extrinsic (i.e., the death receptor pathway) and/or intrinsic apoptotic pathway (i.e., mitochondrial, MOMP-mediated pathway) occur in response to developmental, homeostatic, or internal damage signals [284,285,286,287]. These types of cell death converge in a series of biochemical events including: activation of caspases, cell shrinkage, and plasma membrane blebbing [17,288,289,290].

Necrosis had previously been considered as unregulated and accidental cell death, a toxic process, where the cell is a passive victim that follows an energy-independent mode of death [287,288,291]. Regarding the novel classification, now it is more correct to classify separately MPT-mediated necrosis, which is often caused by intracellular factors, including oxidative stress and Ca^2+^ overload, leading to cell swelling, and eventually to the rupture of the plasma membrane and the typical necrotic morphological changes [283,289,290,291].

**Figure 3:**
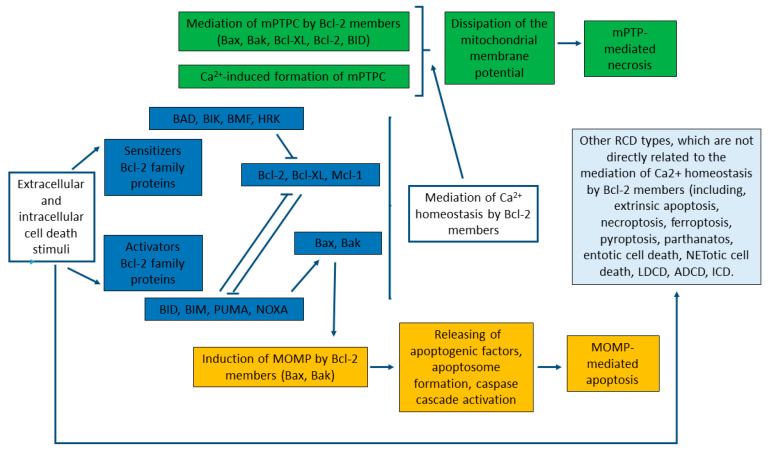
Bcl-2 proteins and mitochondrial Ca^2+^ transport systems determine the necrotic or apoptotic cell death pathways. mPTP-mediated necrosis and MOMP-mediated apoptosis are initiated by various extracellular and intracellular cell death stimuli. Central events for cell death rely on interactions between “activators” and “sensitizers” BH3-only proteins (these proteins are marked by dark blue). Activators (BID, BIM, PUMA, NOXA) bind and activate Bax and Bak and induce a series of their conformational changes and subsequent oligomerization within the OMM, finally resulting in MOMP. Sensitizers (BAD, BIK, BMF, HRK) bind the pro-survival Bcl-2 proteins (Bcl-2, Bcl-XL, Mcl-1) and release them to activate Bax and Bak. MOMP results in the release of pro-apoptogenic factors, formation of an apoptosome, and activation of the cascade that leads to MOMP-mediated apoptosis (these events are marked by orange). In contrast, the central event for mPTP-mediated necrosis is the Ca^2+^-induced formation of mPTPC in the IMM and its mediation by the Bcl-2 members (Bax, Bak, Bcl-XL, Bcl-2, BID). mPTP opening rapidly dissipates the proton gradient across the IMM and induce mPTP-mediated necrosis (all of these events are marked by green). Thus, a possible functional link between Bcl-2 proteins and Ca^2+^-transport systems and between MOMP-mediated apoptosis and mPTP-mediated necrosis may exist. Intracellular or extracellular cell death stimuli may lead to the other types of RCD that are not directly related to the mediation of Ca^2+^ homeostasis by Bcl-2 members (including extrinsic apoptosis, necroptosis, ferroptosis, pyroptosis, parthanatos, entotic cell death, NETotic cell death, LDCD, ADCD, ICD) (these events are marked by light blue).

mPTP-mediated necrosis and mitochondrial-mediated apoptosis are two types of RCD that are mediated through the interconnection of the mitochondrial transport of Calcium and Bcl-2 proteins (Figure 3) [3,214]. Although both of the biochemical mechanisms and morphological changes of apoptosis and necrosis vary significantly, both mechanisms can be characterized as the “apoptosis-necrosis continuum” with a shared biochemical network [292]. Bcl-2 proteins are crucially involved in these processes by working as the key regulators of the MOMP-mediated pathway of apoptosis. This includes MOMP, primarily induced by direct binding interactions between Bcl-2 proteins (Figure 3), leading to irreversible release of intermembrane space proteins, and subsequent caspase activation, which culminates in biochemical and morphological apoptotic changes, and apoptosis itself [218,242,289,293].

As already outlined in Chapter 4, Bcl-2 proteins also interact with mPTP, which represents the key component in mPTP-mediated necrosis. Thus, the interactions between Bcl-2 proteins and mitochondrial Ca^2+^ transport systems might be crucial for determination of the cell death pathway—either the apoptotic or necrotic one [3,292,294] (Figure 3). Of note, mPTP opening originally was solely associated with early apoptosis; however, it was recently shown that MOMP, mediated by mPTP, is linked more closely to the late events of apoptosis and mPTP-mediated necrosis [3,174,295].

The crucial event criterion of mPTP-mediated necrosis is that Ca^2+^-induced formation of mPTPC takes place in the IMM [175,296]. This process is relatively quick (usually taking minutes) and is a result of an elevated Ca^2+^ concentration in the mitochondrial matrix [259]. Bcl-2 proteins are crucially involved in the mediation of mPTP opening, which rapidly dissipates the proton gradient across the IMM required for ATP synthesis [3,169,174,258]. Similarly, it has been demonstrated that the pro-apoptotic Bax and Bak function as the mPTPC components in the OMM and could regulate the necrotic cell death [258]. More specifically, loss of Bax/Bak decreased OMM permeability without changing IMM MPTPC function, leading to resistance to mitochondrial Ca^2+^-overload and mPTP-mediated necrosis. Furthermore, it has been shown that membrane reconstitution of mutants of Bax with their propensity to oligomerize and form disabled membrane pores, yet still capable of enhancing OMM permeability, allowed mPTPC-dependent swelling of mitochondria and restored necrotic cell death [258]. It has also been suggested that Bax and Bak are able to mediate mPTP opening through interactions between OMM and IMM protein contact sites [156].

Interestingly, anti-apoptotic Bcl-2 members can also mediate the activity of mPTP by direct interactions with regulatory components [169]. For instance, Bcl-XL was once thought to be present exclusively on the OMM, but currently it is widely accepted as a F0F1-ATP synthase regulator in the IMM [281,297]. Moreover, pharmacological inhibition of Bcl-2 and/or BCL-XL may induce cell sensitivity to mPTP-mediated necrosis [298]. BID has been characterized as the BH3 protein, which could cooperate with the mPTPC components and regulate necrosis [299]. Altogether, both mPTP-mediated necrosis and MOMP-induced apoptosis can be regulated by Bcl-2 proteins, and triggered by mitochondrial Ca^2+^ overload; causing damage of IMM integrity with catastrophic consequences, particularly bioenergetic collapse by dissipation of the mitochondrial membrane potential (Figure 3) [3,32,283,293].

## 6. Conclusions

In this paper, we have tried to outline a possible functional link between Bcl-2 proteins and calcium transport systems in mitochondria. The ability of Bcl-2 to control Ca^2+^ at multiple levels plays an undisputed role in mediation of Ca^2+^ homeostasis and cell death pathways. MOMP-mediated apoptosis and mPTP-mediated necrosis are determined by Ca^2+^ homeostasis in cells, and particularly in the mitochondria. In this respect, the opening of mPTP and MOMP are interconnected functionally through mitochondrial Ca^2+^ transport systems, and both lead to cell death—either MOMP-mediated apoptosis or mPTP-mediated necrosis.

Furthermore, there exists a regulatory dualism between Bcl-2 proteins and Ca^2+^ transport systems in mitochondria: Bcl-2 proteins not only mediate the activity of mitochondrial Ca^2+^ transport systems, but at the same time the changes in Ca^2+^ transport are important for the induction of mitochondrial cell death pathways via Bcl-2 proteins. Great efforts have been made to understand the interactions of Bcl-2 proteins and their ability to mediate cell fate by regulation of Ca^2+^ homeostasis. However, the regulatory mechanisms of these interactions are still an open issue, which requires additional research. Undoubtedly, a detailed understanding of mitochondrial processes is very important for the analysis of the functions and dysfunctions in living cells, from the viewpoint of mitochondrial biochemistry as well as from the viewpoint of applications in mitochondrial biomedicine used for the diagnosis and treatment of various complex diseases at the subcellular and molecular levels.

## Figures and Tables

**Table 1:** The interactions between mitochondrial Ca^2+^ transport systems and Bcl-2 proteins.

Direction of Ca^2+^ Transport	Mitochondrial Membrane Through Which Ca^2+^ Transport is Realized	The System Responsible for Ca^2+^ Transport	Characteristic Features of the Transport System	Regulation of Mitochondrial Ca^2+^ Transport Systems by Bcl-2 Proteins
Ca^2+^ influx	OMM	VDAC	The main transport system for metabolites, cations, and anions across OMM. It serves as a contact point between the OMM and IMM [42]; 3 different VDAC isoforms have been identified [45,48,53,60]	Ca^2+^ influx via VDAC is regulated by Bcl-2, Bcl-XL and Mcl-1 [29,254,255,256,257,260,263]
	IMM	MCU	MCU is the major pathway of the mitochondrial Ca^2+^ uptake [9,17,60,63,64,65]. MCU consists of several subunits (see Box 2 for details [65,66,68,69,70]	Activity of MCU is mediated by Bcl-2 proteins (BID, BAD, Bcl-XL) [194].
		RaM	RaM accumulates Ca^2+^ with the kinetics hundreds of times faster than MCU. It could also represent a different form or substrate of MCU [89,91]	
		mRyR (mitochondrial ryanodine receptor)	The isoform of RyR1. Proposed as a Ca^2+^-influx system, involved in the regulation of Ca^2+^ efflux under mitochondrial Ca^2+^ overload [92,93]	
		LETM1	Proposed as Ca^2+^/H^+^ antiporter, also involved in the K^+^/H^+^ exchange. Shares a key role with MCU in catalyzing Ca^2+^ uptake into mitochondria [62,96,101,103]	
Ca^2+^ efflux	OMM	VDAC	See above	See above
		mPTP pore complex	ANT, PiC and CypD serve as mPTP regulators in the IMM. The F1F0ATP synthase has been suggested as pore-forming unit in the IMM [110,135,138,141,144,148]	Activity of mPTPC is mediated by the ensemble of Bcl-2 proteins (Bax/Bak, BID, BAD, Bcl-XL) [255,258,259,261]
	IMM	(NCLX) Na^+^/Ca^2+^/Li^+^ exchanger	Na^+^-dependent Ca^2+^ transport also includes the transport of Li^+^. It is typical for excitable tissues (brain, heart) [113,114].	Bcl-2 protein can modulate the activity of NCLX [264].
		HCX (H^+^/Ca^2+^ exchanger)	Na^+^-independent Ca^2+^ efflux is dominant in the non-excitable tissues (liver, kidney, lung, smooth muscle) [119,126]	
		LETM1	Proposed as an alternative mechanism for the regulation of Ca^2+^ release and functions as a Ca^2+^/H^+^ antiporter during specific conditions [62,96,101,102,103]	
		mPTP pore complex	See above	See above

## Abbreviations

ATP	adenosine triphosphate
Bcl-2	B-cell lymphoma-2
TCA	tricarboxylic acid
MOMP	mitochondrial outer membrane permeabilization
NADH	nicotinamide adenine dinucleotide
IMM	inner mitochondrial membrane
IMS	intermembrane space
OMM	outer mitochondrial membrane
MCU	mitochondrial Ca^2+^ uniporter
CC-domains	coiled-coil domains
mRyR	mitochondrial ryanodine receptor
RaM	rapid mode of Ca^2+^ uptake
LETM1	leucine zipper- EF-hand containing transmembrane protein
ETC	electron transport chain
NCX	Na^+^/Ca^2+^ exchanger
NCLX	Na^+^/Ca^2+^/Li^+^ exchanger
HCX	H^+^/Ca^2+^ exchanger
mPTPC	mitochondrial permeability transition pore complex
mPTP	mitochondrial permeability transition pore
ANT	adenine nucleotide translocator
CypD	cyclophilin D
PiC	phosphate carrier
VDAC	voltage-dependent anion channel
MCUb	mitochondrial Ca^2+^ uniporter dominant negative beta subunit
EMRE	essential MCU regulatory element
MICU	mitochondrial Ca^2+^ uptake protein
MCUR1	mitochondrial Ca^2+^ uniporter regulator 1
RaM	RApid mode of Ca^2+^ uptake
SLC25A23	solute carrier 25A23
mRyR	mitochondrial ryanodine receptor
PiC	phosphate carrier
SPG7	m-AAA protease Spastic Paraplegia 7
Mff	mitochondrial fission factor
BH domains	Bcl-2 homology domains
Bcl-XL	Bcl-extra long
Mcl-1	myeloid cell leukemia-1
Bcl-w	Bcl-2-like protein 2
BFL-1/A1	Bcl-2-related protein A1
BIM	Bcl-2-like 11
BID	BH3 interacting domain death agonist
PUMA	p53-upregulated modulator of apoptosis
NOXA	phorbol-12-myristate13-acetate-induced protein 1
BAD	Bcl-2-associated agonist of cell death
BIK	Bcl-2 interacting killer
HRK	Harakiri protein
HRK/DP5	Harakiri protein/Bcl-2 interacting protein death protein 5
BMF	Bcl-2 modifying factor
SMAC	second mitochondria-derived activator of caspase
DIABLO	direct IAP binding protein with low pI
HtrA2	high temperature requirement protein A2
Mfn	mitofusin
ROS	reactive oxygen species
RCD	regulated cell death
PCD	programmed cell death
ADCD	autophagy-dependent cell death
ICD	immunogenic cell death
LDCD	lysosome-dependent cell death
ER	endoplasmic reticulum
SR	sarcoplasmic reticulum
